# Measuring colorectal cancer incidence: the performance of an algorithm using administrative health data

**DOI:** 10.1186/s12874-018-0494-x

**Published:** 2018-05-08

**Authors:** Mamadou Diop, Erin C. Strumpf, Geetanjali D. Datta

**Affiliations:** 1 Department of Social and Preventive Medicine, École de santé publique, Université de Montréal , C.P. 6128, succursale Centre-ville, Montreal, QC H3C3J7 Canada; 2 Research Center of the Centre Hospitalier de l’Université de Montréal (CRCHUM), Tour St Antoine, 850, rue St-Denis, Office 03-456, Montreal, QC H2X0A9 Canada; 3 Department of Economics and Department of Epidemiology, Biostatistics and Occupational Health, McGill University, Leacock Building, Room 418, 855 Sherbrooke Street West, Montreal, QC H3A 2T7 Canada; 4 Direction régionale de santé publique du CIUSSS du Centre-Sud-de-L’Île de Montréal, 1301 Sherbrooke St East, Montreal, QC H2L 1M3 Canada

**Keywords:** Colorectal cancer, Algorithm, Incidence, Administrative health data, Cancer registry

## Abstract

**Background:**

Certain cancer case ascertainment methods used in Quebec and elsewhere are known to underestimate the burden of cancer, particularly for some subgroups. Algorithms using claims data are a low-cost option to improve the quality of cancer surveillance, but have not frequently been implemented at the population-level. Our objectives were to 1) develop a colorectal cancer (CRC) case ascertainment algorithm using population-level hospitalization and physician billing data, 2) validate the algorithm, and 3) describe the characteristics of cases.

**Methods:**

We linked physician billing, hospitalization, and tumor registry data for 2,013,430 Montreal residents age 20+ (2000–2010). We compared the performance of three algorithms based on diagnosis and treatment codes from different data sources. We described identified cases according to age, sex, socioeconomic status, treatment patterns, site distribution, and time trends. All statistical tests were two-sided.

**Results:**

Our algorithm based on diagnosis and treatment codes identified 11,476 of the 12,933 incident CRC cases contained in the tumor registry as well as 2317 newly-captured cases. Our cases share similar overall time trends and site distributions to existing data, which increases our confidence in the algorithm. Our algorithm captured proportionally 35% more individuals age 50 and younger among CRC cases: 8.2% vs. 5.3%. The newly captured cases were also more likely to be living in socioeconomically advantaged areas.

**Conclusions:**

Our algorithm provides a more complete picture of population-wide CRC incidence than existing case ascertainment methods. It could be used to estimate long-term incidence trends, aid in timely surveillance, and to inform interventions, in both Quebec and other jurisdictions.

**Electronic supplementary material:**

The online version of this article (10.1186/s12874-018-0494-x) contains supplementary material, which is available to authorized users.

## Background

Cancer is a leading cause of death in North America [1–3] and colorectal cancer (CRC) is the second most common cause of cancer death in Quebec, Canada. Accurate cancer surveillance is necessary for appropriate resource allocation and to understand the impacts of improvements in screening and treatment on population health. However, accurate surveillance continues to be a challenge in many North American jurisdictions [4], limiting decision makers’ ability to understand the full scope of cancer’s disease burden and to plan accordingly.

The Quebec tumor file was the primary source of cancer surveillance data in the province from 1961 to 2011 [5]. Cancer cases were ascertained using principally diagnostic codes from hospitalization data, with some additional cases ascertained from death certificates and information provided by other jurisdictions if a Quebec resident was treated outside of the province [6]. These data are known to underestimate the burden of cancer, especially among sub-groups who may receive treatment without being admitted to a hospital, such as those with early, less invasive disease [6]. Previous studies have reported incomplete case ascertainment of incident colorectal cancer from administrative data when hospitalization data alone are used [7–9]. If supplementary data, such as treatment codes, are not utilized, hospitalization and physician billing codes can also capture false positive cases, due to detection of prevalent cancers or identifying patients for whom a cancer diagnosis is recorded while a potential cancer diagnosis is being evaluated [10, 11].

Algorithms using administrative health data (medical claims) are one promising avenue to improve the quality of cancer surveillance. Validated algorithms, using “gold standard” comparison groups, have been shown to be representative of the general population and to provide a level of specificity that can permit the identification of cancer cases [12]. Relative to resource-intensive case ascertainment using pathology reports or active reporting by physicians, these algorithms are also low cost and have shorter update delays than cancer registry data [11]. Algorithms based solely on hospitalization data generally display low sensitivity but a high positive predictive value [10, 13–15]. Several authors have demonstrated that the addition of physician billing data can improve the overall performance of cancer case detection algorithms [7, 10–12]. Best practices to ensure completeness of case ascertainment are to use diagnostic and treatment codes in physician billings and other outpatient data sources in addition to hospitalization data [10, 11].

Despite the potential usefulness of administrative data algorithms in cancer surveillance, few studies used them in a population-based setting [9, 15, 16]. Their use has largely been limited to SEER-Medicare data, and therefore among patients age 65 and over, or to single private insurers [17–19]. Colorectal cancer incidence is increasing among patients under age 50 [20], an example that illustrates the need for consistent cancer surveillance tools among younger adults. In the case of Quebec, the cancer surveillance system has undergone a reform in order to improve the exhaustivity and validity of cancer case ascertainment by adding pathology report assessment [6]. Thus, cancer incidence will not be measured consistently over time and an administrative data algorithm will allow the accurate and consistent measurement of long-term trends in cancer incidence. This is particularly timely as the province anticipates instituting an organized CRC screening program in 2018, and it will be important to assess if the program influences changes in cancer incidence.

In this analysis, we 1) develop a new CRC case ascertainment algorithm using diagnosis and treatment data from administrative hospitalization and physician billing data that encompass the entire relevant population, 2) validate the new algorithm using the site distribution and time trends, and 3) describe the differences in case ascertainment completeness according factors such as age and socioeconomic status. Our contributions include measuring and characterizing CRC incidence in the entire Quebec population, using a tool that can be translated to other jurisdictions and can be used to produce consistent cancer incidence estimates over time at little additional cost.

## Methods

### Data sources

Our analyses used population-based, insurance billing data from Quebec’s provincial public insurer, the *Régie de l’assurance maladie du Québec* (RAMQ). The RAMQ insures all physician and hospital services for about 96% of the Quebec population [21] and outpatient prescription drugs for approximately 36% (largely elderly and low-income residents) [22]. Our database includes 2,013,430 Montreal residents age 20 years or older who utilized health services between April 1, 2000 and March 31, 2010 (fiscal years 2000/01–2009/10).

The following data files were linked using an anonymized individual patient identifier: physician fee-for-service billings, hospital admissions, individual death records from the Quebec Statistical Institute (*Institut de la Statistique du Québec*), and the Quebec tumor registry (*Fichier des tumeurs du Québec* - FiTQ). Patients who are admitted to hospital appear in the hospital admissions data. Physician billings include services provided in both inpatient and outpatient settings. Day surgeries can appear in either the hospital admission or the physician billing data, depending on the location of the surgery and if the patient was admitted to the hospital.

### Variables

Like other medical claims databases, the RAMQ data detail health care services received by patients: outpatient visits, hospital admissions, emergency department visits, day surgeries, and billable services (e.g., colonoscopies). The relevant diagnostic (ICD 9 and ICD 10), treatment [23], and procedure codes [24] are included in these data. They also contain information on individual-level demographic characteristics (age, sex, mortality) and small-area measures of socioeconomic status (SES) (Pampalon index of material deprivation [25]).

### Algorithms

**Fig. 1:**
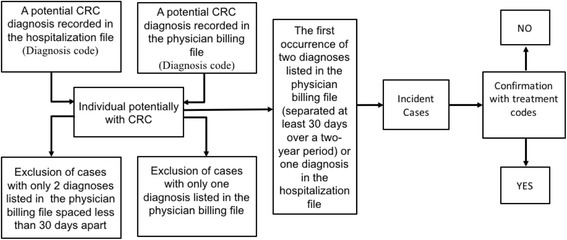
Algorithm to identify incident colorectal cancer cases using administrative health data

We created three algorithms to identify cases of CRC, based on varying source data. Algorithm 1 classified patients with at least one CRC diagnostic code in the hospitalization data as an incident case of CRC. Algorithm 2 classified patients with two diagnostic codes in the physician billing data separated by at least 30 days in a 2-year period, as an incident case of CRC. Algorithm 3 classified patients who meet the criteria under Algorithm 1 and/or 2 as an incident case. A case identified via algorithm 2 but not algorithm 1 would be an individual diagnosed and treated in outpatient settings only. The date of diagnosis was considered the date of admission (algorithm 1), the date of the first of the two diagnoses (algorithm 2), or whichever is first (algorithm 3) (Fig. 1). Relevant diagnostic codes are listed in Additional file 1. We investigated the receipt of surgical, medical, or other colorectal cancer related treatment at any point during our study period among all possible cases (see Additional file 1). Several validation studies of cancer incidence algorithms based on administrative data have demonstrated that the PPV of algorithms utilizing only hospitalization and physician billing data is relatively low [10–12]. Thus, in an effort to improve PPV, the integration of treatment codes in such algorithms has become common and we judged cases to be “true positives” only if the patient met both diagnostic and treatment criteria.

### Statistical analyses

We considered the cases identified in the FiTQ as our reference point, and classified cases as concordant (individuals identified in both the FiTQ and by each of our algorithms) or newly captured cases (individuals identified by our algorithms but not in the FiTQ). We conducted descriptive analyses to compare results from the three algorithms and to select the best performing among them. We selected the algorithm that performed best based on maximizing concordance with the FiTQ and maximizing the number of cases ascertained.

We used two approaches to assess the performance of our algorithm. First, we compared the overall proportion of colon and rectal cancers detected by our algorithm to that documented elsewhere. Second, we compared the trends in age-adjusted incidence rates over time between the FiTQ and our algorithm. We expected that the algorithm would detect a consistently greater number of cases than the FiTQ, but that similar trends over time would indicate the algorithm was detecting true positives. Because we do not have another data source that we consider a valid “gold standard”, we did not assess the performance of our algorithm with measures such as sensitivity and specificity.

To characterize individuals with incident CRC who were not identified in the FiTQ, we compared the proportions of age, sex, socioeconomic status, disease site, and treatment received in the concordant and newly captured cases. We calculated 95 % confidence intervals (CIs) to make comparisons across groups. All statistical test were two-sided and assessed at the *p* < 0.05 level.

Use of the data was authorized by the *Commission d’accès à l’information du Québec*. The study was approved by the Université de Montréal ethics committee (Project 17–033-CERES-D).

## Results

Between 2000 and 2010, 12,933 incident cases of colorectal cancer were captured by the FiTQ (Table 1). Algorithm 1 captured 12,949 cases: 12,930 were concordant with the FiTQ and 19 were newly captured cases. Algorithm 2 captured 13,899 cases: 9940 were concordant with the FiTQ and 3959 were newly captured cases. Algorithm 3 captured 16,897 cases: 12,932 were concordant with the FiTQ and 3965 were newly captured cases. Among identified cases, 11.3% of FiTQ cases did not receive treatment. Among algorithms 1, 2, and 3, the corresponding rates were 11.3%, 14.6%, and 18.4% respectively (Table 1). Considering only treated cases, Algorithm 3 captures 13,793 cases, 11,476 of which are concordant with the FiTQ and 2317 of which are newly-captured (20.2% more). We sought to maximize both concordance and colorectal cancer case ascertainment, thus we selected algorithm 3 as our preferred algorithm on which we conducted further analyses.

**Table 1:** Concordance of incident cases of CRC between the FiTQ and examined algorithms (2000–2010)

Possible Incident CRC Cases		No Treatment Received			Treatment Received			
	Total	Cases concordant with FiTQ	Newly-captured cases	Total	Cases concordant with FiTQ	Newly-captured cases	Total	% Difference from FiTQ
	N	N	N	N	N	N	N	
FiTQ	12,933	1457	–		11,476	–		
Algorithm 1	12,949	1456	2	1458	11,474	17	11,491	0.1
Algorithm 2	13,899	392	1640	2032	9548	2319	11,867	3.4
Algorithm 3	16,897	1456	1648	3104	11,476	2317	13,793	20.2

**Fig. 2:**
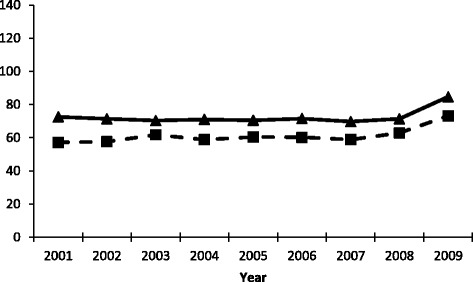
Age-Adjusted CRC Incidence Rates* by Data Sources:  FiTQ  Algorithm *Per 100,000, Quebec population in 2006 used as denominator. NB: Data series truncated due to fiscal to calendar year conversion and the 2-year inclusion criteria

Between 2000 and 2010 age-adjusted incidence rates for CRC were stable, with a small increase at the end of the period (Fig. 2). The rates calculated using algorithm 3 were consistently higher than, and parallel to, those calculated using the FiTQ. The proportion of cases diagnosed as colon cancer in comparison to rectal cancer was similar across the concordant and newly captured cases (Table 2). Approximately 67% of both concordant and newly captured cases were colon cancer cases and approximately 33% were rectal cancers, which is the similar to the distribution reported elsewhere [26, 27]. A very small number (less than 0.22%) did not have a specified disease site. Incident cases detected by our algorithm are similar in both site distribution and overall time trends to existing estimates, increasing our confidence that the algorithm is performing well.

**Table 2:** Characteristics of detected cases: sex, age, socioeconomic status, disease site, and treatment received

	Total		Concordant		Newly-captured cases		Concordant		Newly-captured cases	
			Surgery				Chemotherapy/Radiotherapy			
	N	% (95% CI)	N	% (95% CI)	N	% (95% CI)	N	% (95% CI)	N	% (95% CI)
Total	13,793	100.0	9896	86.2 (85.6 to 86.8)	855	36.9 (34.9 to 38.9)	7807	68.0 (67.2 to 68.9)	1796	77.5 (75.8 to 79.2)
Sex										
Female	6762	49.0 (48.2 to 49.8)	4861	87.0 (86.1 to 87.9)	438	37.3 (34.6 to 40.1)	3600	64.4 (63.2 to 65.7)	912	77.8 (75.4 to 80.1)
Male	7031	51.0 (50.2 to 51.8)	5035	85.5 (84.6 to 86.4)	417	36.5 (33.7 to 39.2)	4207	71.5 (70.3 to 72.6)	884	77.3 (74.8 to 79.7)
Age										
< 50	797	5.8 (5.4 to 6.2)	515	84.8 (82.0 to 87.7)	59	31.1 (24.5 to 37.6)	483	79.6 (76.4. to 82.8)	157	82.6 (77.2 to 88.0)
50–69	4748	34.4 (33.6 to 35.2)	3385	87.3 (86.3 to 88.4)	322	37.0 (33.8 to 40.2)	2928	75.5 (74.2 to 76.9)	686	78.8 (76.0 to 81.5)
70+	8248	59.8 (59.0 to 60.6)	5996	85.8 (84.9 to 86.6)	474	37.7 (35.1 to 40.4)	4396	62.9 (61.7 to 64.0)	953	75.9 (73.5 to 78.2)
Material Deprivation Index										
Q1. Most privileged	2941	21.9 (21.2 to 22.6)	2084	87.0 (85.7 to 88.4)	177	32.4 (28.5 to 36.3)	1609	67.2 (65.3 to 69.1)	444	81.3 (78.1 to 84.6)
Q2	2686	20.0 (19.3 to 19.9)	1909	85.9 (84.4 to 87.3)	158	34.1 (29.8 to 38.4)	1545	69.5 (67.6 to 71.4)	364	78.6 (74.9 to 82.4)
Q3	2752	20.5 (19.3 to 20.7)	1987	86.9 (85.5 to 88.3)	177	38.1 (33.6 to 42.5)	1598	69.9 (68.0 to 71.8)	349	75.1 (71.1 to 79.0)
Q4	2822	21.0 (19.8 to 21.2)	2046	85.5 (84.1 to 86.9)	170	39.5 (34.9 to 44.2)	1642	68.7 (66.8 to 70.5)	319	74.2 (70.1 to 78.3)
Q5. Most deprived	2250	16.7 (16.1 to 17.3)	1621	85.8 (84.2 to 87.4)	154	42.7 (37.6 to 47.8)	1264	66.9 (64.8 to 69.0)	277	76.7 (72.4 to 81.1)
Site*										
Colon	9176	66.5 (65.7 to 67.3)	6756	87.0 (86.3 to 87.8)	532	37.6 (35.1 to 40.1)	4910	63.3 (62.2 to 64.3)	1067	75.5 (73.2 to 77.7)
Rectum	4600	33.4 (32.6 to 34.2)	3138	84.7 (83.6 to 85.9)	321	35.8 (32.7 to38.9)	2886	77.9 (76.6 to 79.3)	724	80.7 (78.1 to 83.3)

Percentages should be interpreted as the proportion of the super column and row that receive the indicated treatment. For example, among concordant cases, 68.0% receive chemo- or radiotherapy. Among concordant cases, 87.0% of women receive surgery. Among newly-captured cases, 75.5% of patients with colon cancer receive chemo- or radiotherapy. Some patients receive both surgery and chemo- or radiotherapy, so the 4 right-most columns do not sum to the total. *The proportion of total cases with unknown site is 0.22%

**Fig. 3:**
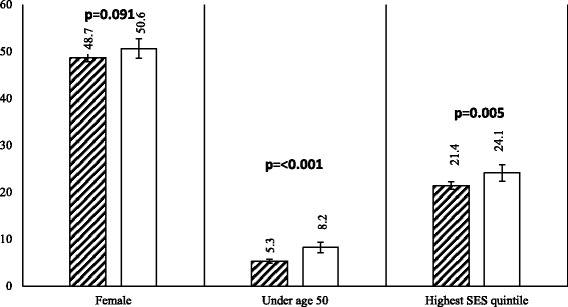
Characteristics of Incident Cases:  concordant vs  newly captured cases

Our algorithm captured a statistically significantly 35.4% greater proportion of people under age 50 among those diagnosed with colorectal cancer relative to the FiTQ: 8.2% (CI_95%_, 7.1% - 9.3%) vs 5.3% (CI_95%_ 4.9% - 5.7%) (Fig. 3). We found approximately equivalent proportions of women and men in our newly captured cases: 50.6% (CI_95%_ 48.6% - 52.7%) vs. 48.7% (CI_95%_ 47.8% - 49.6%). The algorithm captured a statistically significantly higher proportion of cases among people who live in higher SES neighborhoods than the FiTQ: 24.1% (CI_95%_ 22.3% - 25.9%) vs. 21.4% (CI_95%_ 20.6% - 22.2%). These differences in sociodemographic characteristics between the concordant and newly captured cases suggest that FiTQ case ascertainment methods systematically undercount younger patients and those with higher SES.

Treatment patterns by patient characteristics also vary between concordant and newly-captured cases. Among concordant cases, 64.4% of women receive chemo- or radiotherapy (CI_95%_ 63.2% – 65.7%) compared to 71.5% of men (CI_95%_ 70.3% – 72.6%), a statistically significant difference (Table 2). Among newly-captured cases, there is no difference in the proportion of men and women receiving chemo- or radiotherapy. While there are no differences in the proportion of concordant cases receiving surgery by socioeconomic status, among newly-captured cases we see that patients in the most privileged areas are statistically significantly less likely to receive surgery (32.4% CI_95%_ 28.5% – 36.3%) than those in the most deprived areas (42.7% CI_95%_ 37.6% – 47.8%). Among concordant cases, a statistically significantly lower proportion of cases age 70 and above received chemo- or radiotherapy compared to people younger than age 50: 62.9% (CI_95%_ 61.7% - 64.0%, age 70+) vs. 79.6% (CI_95%_ 76.4% - 82.8%, age < 50). While this difference persists in the newly-captured cases, it is no longer statistically significant. These results show that the treatment profiles by sex, socioeconomic status, and age vary between the newly captured and concordant cases.

## Discussion

In this analysis, we show that our algorithm using both diagnosis and treatment information from hospitalization and physician billing data identifies 20% more treated cases of colorectal cancer than methods using only inpatient data. Approximately 11.3% of FiTQ cases and 18.4% of cases detected using only diagnostic information (Algorithm 3) cannot be confirmed with receipt of any treatment. Rates of surgical, chemotherapy and radiation therapy treatment, among cases captured by Algorithm 3 are consistent with rates reported in Canada in the same time period [3, 28]. Our ability to replicate the aggregate time trends in incident CRC over the 2000–2010 period and the typical proportions of colon and rectal cancers also strengthens our confidence in the algorithm’s performance.

In addition to undercounting the number of incident CRC cases, case detection methods that rely only on hospital-based records appear to systematically undercount certain population subgroups. Patients under age 50 and those living in areas with higher socioeconomic status are over-represented in the newly captured cases, relative to cases included in the FiTQ. As those with higher SES have been shown to be diagnosed at earlier stages of disease [29] and those who are diagnosed younger than age 50 are often diagnosed at later stages of disease [30], the newly captured cases are likely to be mixed in terms of stage. Additionally, like previous studies [10, 11], we find that utilizing hospitalization and physician billing data detects a number of cases who cannot be confirmed to have received treatment, which in validation settings are considered false positive cases. Algorithms to detect cancer using administrative data have also been developed in other contexts, although frequently with the limitation that data exist only for patients aged 65 and above [7, 11, 12, 31]. Our study makes the contribution of including data on patients of all ages. Our incident case estimates among younger patients, particularly those ages 50–74 for whom clinical guidelines recommend CRC screening, provide necessary information for disease surveillance, health resources planning, and organized screening programs.

In addition to our substantive findings that help inform cancer control efforts, our work makes a methodological contribution by creating an algorithm that can be replicated and used in the other jurisdictions that have similarly-structured administrative data. This provides a low-cost way to produce cancer incidence statistics using existing data. Our ability to “validate” cases using evidence that treatment was received adds confidence that this is also an effective way to conduct cancer surveillance. This algorithm is particularly valuable in the Quebec because it permits the accurate and consistent measurement of colorectal cancer incidence over time, in the context of recent changes in case ascertainment methods [6].

We recognize that our conservative approach in restricting our case definition to include receipt of treatment may not be ideal in all cases. For example, it is reasonable to expect that some elderly patients diagnosed with colorectal cancer do not pursue surgical, chemo or radiotherapy treatment. While we expect that most of our detected cases without treatment reflect rule-out diagnoses, some of them may indeed be true cancer cases. Depending on the intended purpose, the algorithm could be used in different ways. If policymakers or researchers prefer an inclusive definition at the risk of false positives, case identification based only on diagnostic codes would provide 30% more cases than the FiTQ. On the other hand, if a more specific definition is desired, our selected algorithm offers a promising approach. An even more conservative approach would be to restrict the case ascertainment to only those who has received treatment via surgery, as those cases have been found to have the lowest false positive rates [32–34].

Our study has some inherent limitations, mostly linked to our use of administrative health data. Such data are primarily used to pay providers, and are not designed for disease surveillance. They therefore lack certain details – notably cancer stage – which would facilitate case validation. Dates of diagnosis are often measured with some error in administrative data, and it is difficult to identify the specific physician who actually made the diagnosis, or their specialty. The earliest diagnosis in administrative data has been shown to coincide quite closely with the diagnosis date in clinical databases [35], therefore limiting our concern about serious measurement error on this front. In the absence of a “gold standard” for cancer incidence rates in Quebec, we were unable to calculate the sensitivity, specificity, positive and negative predictive values for our algorithm. While it would of course be useful to have such statistics, the strong performance of administrative data algorithms in other jurisdictions, such as the United States [7], and our ability to validate our identified cases with evidence of treatment received increases our confidence that they are true cases.

## Conclusion

In conclusion, our algorithm using both hospitalization and physician billing data detects more cases of CRC than the FiTQ. It provides a more complete picture of CRC incidence and all detected cases appear to be valid, based on receipt of treatment. This algorithm could be used in Quebec and in other jurisdictions as a cost-effective way to conduct timely cancer surveillance and to inform screening programs and health care resource planning.

## Additional file

Additional file 1: **Appendix I.** Colorectal Cancer Diagnostic Codes. **Appendix II.** Contains colorectal cancer treatment codes. (DOCX 33 kb)

## Abbreviations

**CRC**: Colorectal cancer
**FiTQ**: *Fichier des tumeurs du Québec*
**ICD**: International Classification of Diseases
**RAMQ**: *Régie de l’assurance maladie du Québec*
**SEER**: Surveillance, Epidemiology, and End Results
**SES**: Socioeconomic status
