# Supplementary material for: Measuring colorectal cancer incidence: the performance of an algorithm using administrative health data
Source: BMC Med Res Methodol. 2018 May 8;18:38. doi: 10.1186/s12874-018-0494-x (PMC5941340; doi:10.1186/s12874-018-0494-x)
Supplement: Supplementary file 1 — Appendix I. Colorectal Cancer Diagnostic Codes. Appendix II. Contains colorectal cancer treatment codes. (DOCX 33 kb) [file 12874_2018_494_MOESM1_ESM.docx]

**Supplemental Materials**

**Appendix I : Colorectal Cancer Diagnostic Codes**

|  | ICD-10-CA * | ICD-9-QC ** |
| --- | --- | --- |
| Colon and Cæcum | C18.0 : Malignant neoplasm of colon, Caecum  C18.1 : Malignant neoplasm of colon, Appendix  C18.2 : Malignant neoplasm of colon, Ascending colon  C18.3 : Malignant neoplasm of colon, Hepatic flexure  C18.4 : Malignant neoplasm of colon, Transverse colon  C18.5 : Malignant neoplasm of colon, Splenic flexure  C18.6 : Malignant neoplasm of colon, Descending colon  C18.7 : Malignant neoplasm of colon, Sigmoid colon  C18.8 : Malignant neoplasm of colon, Overlapping lesion of colon  C18.9 : Malignant neoplasm of colon, unspecified | 1534: Malignant neoplasm of colon, cæcum  1535 : Malignant neoplasm of colon, Appendix  1536 : Malignant neoplasm of colon, Ascending colon  1530 : Malignant neoplasm of colon, Hepatic flexure  1531 : Malignant neoplasm of colon, Transverse colon  1537 : Malignant neoplasm of colon, Splenic flexure  1532 : Malignant neoplasm of colon, Descending colon  1533 : Malignant neoplasm of colon, Sigmoid colon  1538 : Malignant neoplasm of colon, NCA  1539 : Malignant neoplasm of colon, SAI |
| Rectosigmoid junction | C19 : Malignant neoplasm of rectosigmoid junction | 1540 : Malignant neoplasm of rectosigmoid junction |
| Rectum | C20.0 : Malignant neoplasm of rectum | 1541 : Malignant neoplasm of rectum |
| Intestinal tract, digestive organs and peritoneum | C26.0 : Malignant neoplasm of other and ill-defined digestive organs, Intestinal tract, part unspecified | 159.0 Malignant neoplasm of other and ill-defined sites within the digestive organs and peritoneum, Intestinal tract, part unspecified |

# *International Statistical Classification of Diseases and Related Health Problems, 10th Revision, Canada

** International classification of diseases 9th revision adapted for Quebec

# Appendix II : Colorectal Cancer Treatment Codes [1, 2]

## Surgery

## Partial colectomy with abdominoperineal amputation of the rectum and colostomy

- 05160
- 05162
- 05163

## Colostomy

- 05029

## Total colectomy with abdominoperineal amputation of the rectum and ileostomy

- 05233
- 05234
- 05235

## Intestinal resection with anastomosis

- 05141 Ileum terminal, cecum
- 05142 Ileum terminal, cecum, Ascending colon and hepatic angle (Right hemicolectomy)
- 05154 Transverse colon or segment of left colon
- 05164 Left hemicolectomy
- 05232 Total colectomy with ileo-rectostomy
- 05165 Partial colon resection with colostomy and distal rectal closure (Hartman)
- 05166 Total colon resection with ileostomy and closure of the distal rectum

## Total colectomy with rectal mucosa resection, ileal reservoir formation, ileoanal anastomosis and bypass ileostomy

- 05279
- 05280
- 05281

## Cryosurgery or fulguration of a malignant tumor of the rectum

- 05192

## Excision of a villous tumor of the rectum by anal way

- 05182

## Perineal or abdominal excision of a tumor with recurrence of malignant tumor of the rectum

- 05183

## Abdominal-sacral resection of the rectum

- 05246

## Protectomy

- 05240 Previous resection
- 05241 Perineal resection, two-stroke
- Abdominal-perineal resection, with pull-through or colostomy
  - 05242
  - 05243
  - 05244
  - 05283

## Radiation therapy

## Private practice

- 09127 Main visit
- 09131 Main visit of a patient who has completed radiotherapy treatment (30 days or more since last treatment)
- 09165 Consultation (including main visit and consultation supplement)
- 09129 Control visit
- 09133 Control visit of a patient who has completed radiotherapy treatment (30 days or more since last treatment)

## Short-term care Hospital Center:

- Hospitalization
  - 09150
  - 09134
  - 09060
  - 09160
  - 09094
  - 09136
  - 09152
  - 09141
  - 09161
  - 09143
- External
  - 09162
  - 09144
  - 09170
  - 09164
  - 09146

## Long-Term Care Hospital:

- 09147
- 09296
- 09176
- 09148

## Home:

- 09171
- 09172

## Hospital Center:

- 09168
- 15465
- 09201
- 09214
- 09212
- 09202
- 09215

## Chemotherapy

## Follow-up and administration, where appropriate, of intravenous chemotherapy treatments for cancer patients for which the general surgeon is responsible

- 15272

## Follow-up and administration, where appropriate, of intravenous chemotherapy to cancer patients for which the internal medicine specialist is responsible

- 15403

## Oncological follow-up visit including the determination of the chemotherapy treatment and the supervision of it by the neurosurgeon

- 15515

## Patient visit under chemotherapy, graft or immunosuppression primary or secondary

- 15550

## Supervision of chemotherapy administration

- 00734

## Intra-abdominal injection (chemotherapy)

- 00470 The first injection
- 00472 Each subsequent injections at the same session

## Intrapleural injection (chemotherapy)

- 00475 The first injection
- 00476 Each subsequent injections at the same session

## Administration of chemotherapy including therapeutic evacuation and diagnostic blood draw:

- 00583 First injection
- 00603 Each subsequent injection at the same session

1. Régie de l'assurance maladie du Québec. *Présentation de la Régie de l'assurance maladie du Québec: un partenaire dynamique dans la gestion et l'évolution du système de santé québécois*. http://collections.banq.qc.ca/ark:/52327/bs2248355.

2. ICIS, Institut canadien d'information sur la santé. *Classification canadienne des interventions en santé*. https://www.cihi.ca/fr/donnees-et-normes/normes/classification-et-codification/classification-canadienne-des-interventions.
